# Post-hoc selection of dynamic causal models

**DOI:** 10.1016/j.jneumeth.2012.04.013

**Published:** 2012-06-30

**Authors:** M.J. Rosa, K. Friston, W. Penny

**Affiliations:** Wellcome Trust Centre for Neuroimaging, Institute of Neurology, University College London, 12 Queen Square, WC1N 3BG, UK

**Keywords:** Bayesian model selection, Model evidence, Free energy bound, Dynamic causal modelling, FMRI, Connectivity

## Abstract

Dynamic causal modelling (DCM) was originally proposed as a hypothesis driven procedure in which a small number of neurobiologically motivated models are compared. Model comparison in this context usually proceeds by individually fitting each model to data and then approximating the corresponding model evidence with a free energy bound. However, a recent trend has emerged for comparing very large numbers of models in a more exploratory manner. This led Friston and Penny (2011) to propose a post-hoc approximation to the model evidence, which is computed by optimising only the largest (full) model of a set of models. The evidence for any (reduced) submodel is then obtained using a generalisation of the Savage-Dickey density ratio (Dickey, 1971). The benefit of this post-hoc approach is a huge reduction in the computational time required for model fitting. This is because only a single model is fitted to data, allowing a potentially huge model space to be searched relatively quickly. In this paper, we explore the relationship between the free energy bound and post-hoc approximations to the model evidence in the context of deterministic (bilinear) dynamic causal models (DCMs) for functional magnetic resonance imaging data.

## Highlights

► New (post-hoc) procedure for approximating the model evidence is applied to deterministic bilinear dynamic causal models for fMRI. ► Post-hoc procedure is based on optimising only the largest model of a set of models. ► Good agreement found between free energy bound and post-hoc approximations to the model evidence and model parameters. ► Post-hoc approach reduces computational time immensely and can be applied to any nested models.

## 1 Introduction

Bayesian model selection (BMS) is a powerful method to compare different models for explaining observed data. BMS is based on the model evidence, which is the probability of obtaining a particular model, given the data. Even though this quantity is not, in general, straightforward to compute, it is now well established that statistical models can be compared using a variational free energy approximation to the evidence (Beal and Ghahramani, 2003). This approximation has widespread application, and, in neuroimaging, it has become the method of choice for comparing models of effective brain connectivity, in particular dynamic causal models (DCMs) (Stephan et al., 2010; Penny, 2012).

Dynamic causal modelling is a mathematical framework to estimate, and make inferences about, the coupling among brain areas and how this coupling is influenced by changes in experimental context (Friston et al., 2003). Although it was originally introduced as a hypothesis driven procedure, in which a small number of neurobiologically motivated models are compared, recently, a trend has emerged for comparing very large numbers of models in a more exploratory manner.

Model comparison in this context has hitherto proceeded by individually fitting all competing models to data and then approximating the model evidence with the variational free energy bound (Friston et al., 2007). We refer to this approximation to the model evidence as the optimised evidence.

Very recently, Friston and Penny (2011) have proposed an alternative, post-hoc, approximation to the model evidence that is computed by fitting only the very largest of a set of models: a full model from which all other (reduced) models can be formed by removing model parameters. This scheme approximates the evidence for any nested model within a larger model using only the posterior density of the full model. We refer to this approximation as the post-hoc evidence.

The benefit of this post-hoc approach is a huge reduction in the computational time required for model fitting. This is because only a single model is fitted to data. This means that a potentially huge model space can be searched relatively quickly.

In addition to the model evidence approximation, Friston and Penny (2011) also propose a way to estimate the connectivity parameters for all reduced models from the posterior density over the parameters of the full model. More specifically, according to Friston and Penny (2011) the posterior mean and precision of the reduced model can also be determined solely from the mean and precision of the parameters of the full model.

The post-hoc approach (Friston and Penny, 2011), can also be viewed as a generalisation of the well-known Savage-Dickey density ratio (Dickey, 1971), in which the reduced models have certain parameters fixed at zero. To our knowledge, the Savage-Dickey method (Dickey, 1971), has not yet been applied to neuroimaging problems, although it has been applied in other fields, from cognitive psychology (Wagenmakers et al., 2010) to cosmological models (Trotta, 2007). The recently proposed post-hoc approach (Friston and Penny, 2011) has been developed with neuroimaging models in mind, and the authors have shown (Friston et al., 2011), using stochastic DCMs, that there is a very good agreement between the optimised and post-hoc model evidences.

In this paper, we explore the relation between optimised and post-hoc approximations to the model evidence in the context of established practises in neuroimaging. Most studies of brain connectivity apply deterministic (rather than stochastic) DCM to data, under specific experimental paradigms. In addition, the models used in Friston et al. (2011), stochastic DCMs, are linear dynamical models, while most DCMs comprise bilinear dynamics corresponding to modulatory effects on brain connections (i.e., the underlying state-space model is nonlinear in the hidden states). Here we test if the post-hoc method is applicable to (deterministic) bilinear DCMs.

Since the main goal of DCM is to make inferences on the connectivity parameters we also compare the estimates of these parameters obtained with these two approaches. To this end we use synthetic and real functional magnetic resonance imaging (fMRI) data from a previously published study on attention to visual motion (Buchel and Friston, 1997). This is the same dataset used in Friston et al. (2011). Although we use fMRI data, the methods described here can also be applied to other data modalities and statistical models, as long as the models that are compared are nested.

This paper is structured as follows. In Section 2 we review Dynamic Causal Modelling for fMRI. We then focus on model optimisation and different approaches to estimate the model evidence and connectivity parameters. We then present and discuss results from comparing these approaches using synthetic and real fMRI data.

## 2 Methods

In this section we briefly review dynamic causal models (DCM). We then turn to model inversion and scoring. We look at different proxies for the model evidence: the optimised free energy approximation and the computationally less-expensive post-hoc approximation. The former has been the method of choice in the hypothesis led comparison of DCMs, whilst the latter allow for data-led exploration of much larger model spaces. In addition, we compare the estimates for the connectivity parameters obtained with these two approaches. Finally, we revisit how these approximations can be used for Bayesian model selection (BMS).

### 2.1 Dynamic causal modelling

Dynamic causal modelling is a mathematical framework to estimate, and make inferences about, the coupling among brain areas and how this coupling is influenced by changes in experimental context (Friston et al., 2003). It uses differential equations to describe the neuronal activity of interacting cortical regions and a forward model of how this neuronal activity is transformed into an observed response. This framework has been applied to fMRI, Electroencephalographic (EEG) and Magnetoencephalographic (MEG) (Kiebel et al., 2009), as well as Local Field Potential (LFP) data (Moran et al., 2009). Here, we focus on fMRI but the methods described below can also be applied to other data modalities.

Here we consider DCMs for fMRI that employ a deterministic bilinear model for the dynamics at the neuronal level (neurodynamics) and an extended Balloon model for the haemodynamic level. For non-linear, two-state or stochastic DCMs see Stephan et al. (2008), Marreiros et al. (2009), and Friston et al. (2011), respectively. The deterministic bilinear neurodynamics are described by the following multivariate differential equation:

(1) $$\dot{z}(t)=\left(A+\sum_{j=1}^{M}u_{j}(t)B^{j}\right)z(t)+\mathrm{Cu}(t),$$

where the dot notation denotes the time derivative. The variable *z* describes changes in neuronal activity resulting from the sum of three effects. First, the matrix *A* encodes direct, or fixed, connectivity between pairs of regions. The elements of this connectivity matrix are not a function of the input and can represent both unidirectional and bidirectional connections. Second, the elements of *B*^*j*^ represent the changes in connectivity induced by the inputs *u*_*j*_. These condition-specific modulations or bilinear terms are usually the interesting parameters. Third, the matrix *C* encodes the direct influence of each exogenous input *u*_*j*_ on each area.

The overall structure of fixed, *A*, modulatory, *B*, and input, *C*, connectivity matrices constitutes our assumptions about model structure. This in turn represents a scientific hypothesis about the structure of the large-scale neuronal network mediating the underlying cognitive function.

As mentioned above, DCM for fMRI uses the extended Balloon model to describe how changes in neuronal activity give rise to the observed fMRI signals for each region. The full derivation of the model equations can be found in Buxton et al. (1998) and Friston et al. (2000). See also Stephan et al. (2007) for recent developments. In brief, for a particular region, neuronal activity, *z*, causes an increase in a vasodilatory signal, *s*, that is subject to auto-regulatory feedback. Inflow, *f*, responds in proportion to this signal with concomitant changes in blood volume *ν* and deoxyhaemoglobin content *q*:

(2) $$\begin{array}{c} \frac{\mathrm{ds}(t)}{\mathrm{dt}}=z(t)-\frac{s(t)}{\tau_{s}}-\frac{f(t)-1}{\tau_{f}}\\ \frac{\mathrm{df}(t)}{\mathrm{dt}}=s(t)\\ \tau_{0}\frac{d\nu (t)}{\mathrm{dt}}=f(t)-\nu {\left(t\right)}^{1/\alpha }\\ \tau_{0}\frac{\mathrm{dq}(t)}{\mathrm{dt}}=\frac{f(t)}{E_{0}}[1-{\left(1-E_{0}\right)}^{1/f(t)}]-q(t)\nu {\left(t\right)}^{(1-\alpha )/\alpha }. \end{array}$$

The haemodynamic parameters comprise the rate constant of the vasodilatory signal decay, *τ*_*s*_, the rate constant for autoregulatory feedback by blood flow, *τ*_*f*_, transit time, *τ*_0_, Grubb's vessel stiffness exponent, *α*, and the resting oxygen extraction fraction, *E*_0_. For identifiability reasons, only two of these parameters are estimated from the data for each region: *h* = {*τ*_*s*_, *τ*_0_}. The others are set to *τ*_*f*_ = *α* = *E*_0_ = 0.32.

The Blood Oxygenation Level Dependent (BOLD) signal, *y* is then taken to be a static nonlinear function that comprises a volume-weighted sum of extra- and intra-vascular signals:

(3) $$h(q,V)=V_{0}\left[k_{1}(1-q(t))+k_{2}\left(1-\frac{q(t)}{\nu (t)}\right)+k_{3}(1-\nu (t))\right].$$

The factors *k*_1_, *k*_2_ and *k*_3_ are dimensionless but depend on the characteristics of the fMRI recording system. For 1.5 T and TE of 40 ms, *k*_1_ ≅ 7*E*_0_, *k*_2_ ≅ 2 and *k*_3_ ≅ 2*E*_0_ − 0.2. *V*_0_ = 0.02 is the resting blood volume fraction.

The parameters, *θ*, for a bilinear DCM, indexed by *m*, comprise the connectivity matrices as well as the haemodynamic parameters, i.e. *θ* = {*A*, *B*, *C*, *h*}. The priors, *p*(*θ*|*m*), on both the connectivity and haemodynamic parameters are described in Appendix A. In current implementations of DCM, independent of modality, the model parameters are estimated from the data, *y*, using Bayesian methods, and models are compared using the model evidence.

### 2.2 Model evidence

The model evidence, *p*(*y*|*m*), is the probability of obtaining observed data, *y*, given model *m*, belonging to model space *M*. This quantity is at the heart of Bayesian model selection (BMS), but, in general, it is not straightforward to compute, since this computation involves integrating out the dependency on the model parameters, *θ*:

(4) p(y|m)=∫p(y|θ,m)p(θ|m)dθ

Sampling or iterative analytic methods can be used to approximate the above integral. The method of choice to approximate the evidence for DCMs has been the variational free energy approximation (Friston et al., 2007; Stephan et al., 2010). This method involves individually fitting (optimising) each model to data and then approximating the model evidence with a free energy bound. We refer to this approximation as the optimised model evidence. In contrast, Friston and Penny (2011) have proposed a post-hoc approximation to the evidence, which is computed by optimising only the largest of a set of models. This approach can be viewed as a generalisation of the well-known Savage-Dickey ratio (Dickey, 1971). In addition to the model evidence, the post-hoc scheme also provides estimates of the parameters for all reduced models from the full (optimised) model. Below we describe the variational scheme used to optimise DCMs and the two different approaches to approximate the model evidence and parameters (*optimised* and *post-hoc* approximations).

### 2.3 Model optimisation

In Bayesian inference, prior beliefs about parameters, *θ*, are quantified by the prior density, *p*(*θ*|*m*), which is specified using biophysical and dynamic constraints. Inference on the parameters, *θ*, after observing data, *y*, is based on the posterior density *p*(*θ*|*y*, *m*). These densities are related through Bayes’ rule:

(5) $$p(\theta |y,m)=\frac{p(y|\theta ,m)p(\theta |m)}{p(y|m)},$$

where *p*(*y*|*θ*, *m*) is the probability of the data (likelihood) conditioned upon the model and its parameters. The normalisation factor, *p*(*y*|*m*), is the model evidence (Eq. (4)). The posterior density is an optimal combination of prior knowledge and new observations, and provides a complete description of uncertainty about the parameters.

Under Gaussian assumptions, also known as the Variational Laplace (VL) approximation (Friston et al., 2007), the problem of estimating the posterior density reduces to finding its first two moments, the conditional mean *μ* and conditional covariance *C*. The prior density is also assumed to be Gaussian with mean *η* and covariance *Σ* (see Appendix A).

Non-linear deterministic models, such as DCMs, Eq. (1), can be linearised by expanding the observation equation about a working estimate *μ* of the conditional mean:

(6) $$\begin{array}{c} y=h(\theta ,u)+\epsilon \\ h(\theta ,u)\approx h(\mu )+J\cdot (\theta -\mu ), \end{array}$$

such that $J=\frac{\partial h(\mu )}{\partial \theta }$, *r* = *y* − *h*(*μ*) ≈ *J* · (*θ* − *μ*) + *ϵ* and *ϵ* ∼ *N*(0, *C*_*ϵ*_), where the error covariance is assumed isotropic over the fMRI predictions, *C*_*ϵ*_ = *σ*^2^*I*.

Under the Gaussian assumptions mentioned above, this approximation, Eq. (6), yields the following equations for the conditional mean, *μ*, and precision (inverse of covariance), *P* = *C*^−1^, which can be updated recursively in an optimisation scheme, such as VL:

(7) $$\begin{array}{c} P=J^{T}C_{\epsilon }^{-1}J+\Sigma^{-1}\\ \mu =C(J^{T}C_{\epsilon }^{-1}r+\Sigma^{-1}\eta ). \end{array}$$

The variational approximation to the posterior density has been verified for DCM for fMRI using Markov Chain Monte Carlo (MCMC) (Chumbley et al., 2007). These schemes are more computationally intensive but allow one to estimate the posterior density without assuming it has a fixed Gaussian form.

#### 2.3.1 Optimised evidence

As mentioned before, VL updates the moments of the posterior density, *q*(*θ*|*y*, *m*) by maximising the negative variational Free Energy (henceforth ‘free energy’, *F*_*m*_), which provides a lower bound on the log model evidence, log *p*(*y*|*m*), Beal and Ghahramani (2003):

(8) $$\log p(y|m)=F_{m}+\mathrm{KL}(q(\theta )||p(\theta |y,m)).$$

*KL* is the Kullback–Leibler divergence between the approximate and true posterior. This quantity is always positive, or zero when the densities are identical.

It is usually assumed that Eq. (8) is a tight bound such that model comparison can then proceed using *F*_*m*_ as a surrogate for the log-evidence. We call this approximation *optimised evidence* because it comes out of the optimisation scheme described above. The Laplace approximation to the free energy (Friston et al., 2007) yields an estimate, which is not strictly a lower bound on the model evidence (Penny, 2012; Wipf et al., 2010). Nevertheless, it provides a very useful model comparison criterion (Penny, 2012).

Other approximations to the optimised model evidence exist, including the computationally more expensive Annealed Importance Sampling (AIS) method (Beal and Ghahramani, 2003), and the simpler but potentially less accurate Bayesian Information Criterion (BIC) and Akaike Information Criterion (AIC) measures (Penny et al., 2004). In extensive simulations of graphical model structures, Beal and Ghahramani (2003) found that the variational approach outperformed BIC and AIC, at relatively little extra computational cost, and approached the performance of AIS, but with much less computational cost. In addition, Penny (2012) shows that for the comparison of DCMs, the free energy approach also performs better than either AIC or BIC. In this work we use the Laplace approximation to the free energy (optimised) evidence described above.

All these approximations to the model evidence, however, are based on inverting all models in the model space. This is feasible only in a hypothesis driven procedure in which the model space comprises a small number of models. In large model spaces, optimising all models to obtain the evidences rapidly becomes computationally unfeasible. For instance, in more exploratory analyses, one might be interested in looking at most, if not all, the possible connections and modulatory effects. The model space in this case can easily have thousands or millions of different networks. Below, we describe a less computationally expensive alternative to compute the model evidences.

#### 2.3.2 Post-hoc evidence

This approach provides the model evidence and parameters for any nested (reduced) model within a larger (full) model as a function of the posterior density of the full model (Friston and Penny, 2011). This is a flexible approach that allows for post-hoc model selection without the need to invert more than a single model. In DCM the full model may be, for example, the fully connected network and the reduced models would correspond to networks with a sparser connectivity contained within this larger model.

The method assumes only the existence of a full model, *m*_*F*_ ∈ *M*, which shares the same likelihood with the set of reduced models, *m*_*i*_ ∈ *M* and ∀_*i*_ : *m*_*i*_ ≺ *m*_*F*_:

(9) $$p(y|\theta ,m_{i})=p(y|\theta ,m_{F}).$$

This means the reduced models are constructed from the full model only by changing the priors on certain parameters *θ*^*u*^ ⊂ *θ* as described below. This also implicitly assumes that the hyperparameters describing observation noise levels, *λ*_*obs*_, are the same for the full and reduced models. This is not the case for the optimised model evidence approach, where *λ*_*obs*_ are optimised for each model.

We can then use Bayes rule to transform the above equality, Eq. (9). By re-arranging the terms we can write the ratio of model evidences in terms of the posterior and priors of the full and reduced model:

(10) $$\frac{p(y|m_{i})}{p(y|m_{F})}=\frac{p(\theta |y,m_{F})}{p(\theta |y,m_{i})}\frac{p(\theta |m_{i})}{p(\theta |m_{F})}$$

Friston and Penny (2011) consider Eq. (10), under the Laplace approximation, as mentioned above. Under this approximation the posteriors, *q*, and priors, *p*, of the full and reduced models are Gaussian densities:

(11) $$\begin{array}{c} q(\theta |m_{i,F})=N(\mu_{i,F},C_{i,F}):C_{i,F}=P_{i,F}^{-1}\\ p(\theta |m_{i,F})=N(\eta_{i,F},\Sigma_{i,F}):\Sigma_{i,F}=\Pi_{i,F}^{-1}, \end{array}$$

where *η*_*i*,*F*_ and *Π*_*i*,*F*_ are the prior means and precisions for the reduced (*i*) and full model (*F*), while *μ*_*i*,*F*_ and *P*_*i*,*F*_ are the posterior means and precisions. Making use of the assumptions of Eq. (11) in Eq. (10) the log model evidence for any reduced model can be written as a simple analytic function of the means and precisions of the prior and posterior of the full and reduced model:

(12) $$F_{i}=\log p(y|m_{i})=\frac{1}{2}\log\frac{\left|\Pi_{i}||P_{F}\right|}{\left|P_{i}|||\Pi_{F}\right|}-\frac{1}{2}(\mu_{F}^{T}P_{F}\mu_{F}+\eta_{i}^{T}\Pi_{i}\eta_{i}-\eta_{F}^{T}\Pi_{F}\eta_{F}-\mu_{i}^{T}P_{i}\mu_{i})+F^{F}.$$

This is useful because the requisite means and precisions of the reduced model can be derived in a straightforward way from the means and precisions of the full model (see below).

The post-hoc approach can also be viewed as a generalisation of the Savage-Dickey density ratios (Dickey, 1971), in which the reduced models have certain parameters fixed at zero. To obtain these ratios we integrate Eq. (10) over the parameters. To do this we first partition the parameter space into two subsets of parameters *θ* = {*θ*^*u*^, *θ*^*c*^}. The subset *θ*^*u*^ ⊂ *θ* contains all the parameters which differ between the full, *F*, and reduced model, *i*. The remaining parameters *θ*^*c*^ are shared between the models, with equal priors: *p*(*θ*^*c*^|*m*_*i*_) = *p*(*θ*^*c*^|*m*_*F*_). We refer to *θ*^*u*^ and *θ*^*c*^ as the unique and common parameters, respectively, and assume the priors factorise, i.e. *p*(*θ*|*m*_*i*_) = *p*(*θ*^*u*^|*m*_*i*_)*p*(*θ*^*c*^|*m*_*i*_). With this notation, we can write Eq. (10) as follows:

(13) $$\begin{array}{c} \int p(\theta |y,m_{i})\frac{p(y|m_{i})}{p(y|m_{F})}\;d\theta =\int p(\theta |y,m_{F})\frac{p(\theta |m_{i})}{p(\theta |m_{F})}\;d\theta \\ \frac{p(y|m_{i})}{p(y|m_{F})}=\int \int p(\theta |y,m_{F})\frac{p(\theta |m_{i})}{p(\theta |m_{F})}\;d\theta^{u}\;d\theta^{c}, \end{array}$$

where ∫*p*(*θ*|*y*, *m*_*i*_)*dθ* = 1. If we then use *p*(*θ*^*u*^, *θ*^*c*^|*y*, *m*_*F*_) = *p*(*θ*^*c*^|*θ*^*u*^, *y*, *m*_*F*_)*p*(*θ*^*u*^|*y*, *m*_*F*_) and the fact that the priors over *θ*^*c*^ are the same for both models we obtain the following result:

(14) $$\frac{p(y|m_{i})}{p(y|m_{F})}=\int \int p(\theta^{c}|\theta^{u},y,m_{F})p(\theta^{u}|y,m_{F})\frac{p(\theta^{u}|m_{i})}{p(\theta^{u}|m_{F})}\;d\theta^{u}\;d\theta^{c}=\int p(\theta^{u}|y,m_{F})\frac{p(\theta^{u}|m_{i})}{p(\theta^{u}|m_{F})}\;d\theta^{u}.$$

When the reduced prior is a point mass (delta function), $p(\theta^{u}|m_{i})=\delta ({\bar{\theta }}^{u})$, that fixes the subset of parameters *θ*^*u*^ to a particular value, ${\bar{\theta }}^{u}$, the last equation, Eq. (14), reduces to the Savage-Dickey ratio (usually considered when ${\bar{\theta }}^{u}=0$):

(15) $$\frac{p(y|m_{i})}{p(y|m_{F})}=\frac{p(\theta^{u}=0|y,m_{F})}{p(\theta^{u}=0|m_{F})}.$$

**Fig. 1:**
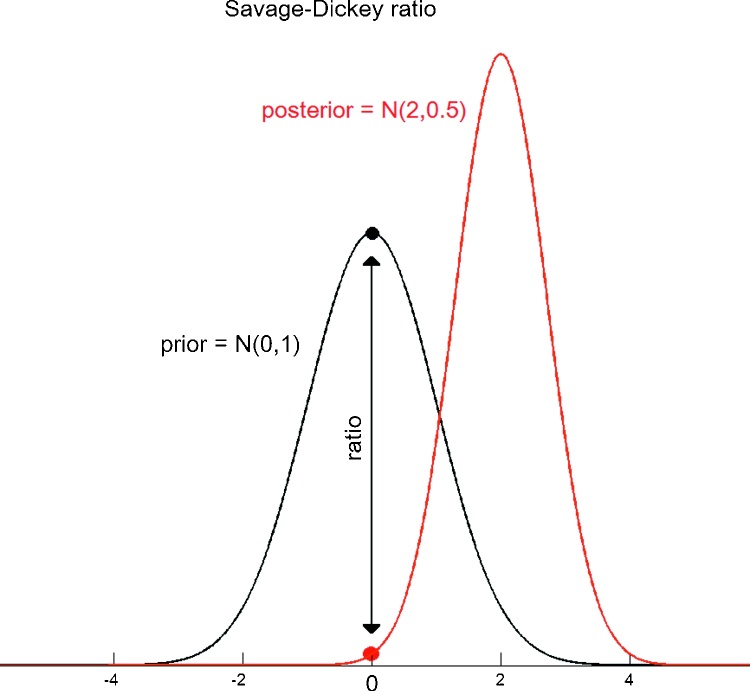
Savage-Dickey density ratio: This ratio is calculated by dividing the value of the posterior distribution over the parameters for the full model evaluated at *θ* = 0, *p*(*θ* = 0|*y*, *m*), by the prior for the same model evaluated at the same point, *p*(*θ* = 0|*m*). These quantities are shown here for the case of univariate Gaussian prior and posterior. The interpretation is very simple: if it is less likely that parameters *θ* equal 0 after seeing the data (posterior) than before (prior), then *p*(*y*|*m*_*i*_)/*p*(*y*|*m*_*F*_) < 1 and we have evidence in favour of the full model, *m*_*F*_, and vice-versa.

This ratio has a simple intuitive interpretation: if we believe it is more likely that parameters *θ*^*u*^ are zero after seeing the data than before, then *p*(*y*|*m*_*i*_)/*p*(*y*|*m*_*F*_) > 1 and we have evidence in favour of the reduced model *m*_*i*_. This is depicted in Fig. 1.

The posterior of the full model can again be obtained using the VL optimisation scheme, *q*(*θ*|*y*, *m*_*F*_), described above. Again under Gaussian assumptions we can write the previous ratio, Eq. (15), as follows:

(16) $$F_{i}^{u}=\log p(y|m_{i})=\frac{1}{2}\log\frac{\left|P_{F}^{u}\right|}{\left|\Pi_{F}^{u}\right|}-\frac{1}{2}(\mu_{F}^{u^{T}}P_{F}^{u}\mu_{F}^{u}-\eta_{F}^{u^{T}}\Pi_{F}^{u}\eta_{F}^{u})+F_{F}^{u}.$$

This analytic formula is a special case of the post-hoc approach, Eq. (12), to calculate the model evidence of any reduced model as a function solely of the posterior mean and precision of the full model. The difference between Eq. (12) and Eq. (16) is the absence of quantities from the reduced model and the fact that all means and precisions are taken only for the subset of unique parameters, *θ*^*u*^, which are not allowed to vary in the reduced model.

#### 2.3.3 Post-hoc parameters

Once the full model has been optimised, Eq. (12) can be used to compute the model evidences for all reduced models from the full model. This results from the fact that, as we describe in the following, the posterior mean and precision of the reduced model parameters can also be determined from the mean and precision of the full model.

To obtain these estimates we again assume that the models differ only in the specification of the priors, i.e. they share the same likelihood, Eq. (9). Using this assumption we can subtract the linearised approximation to the conditional precision, Eq. (7), of the full model from the precision of the reduced model and eliminate the terms that do not depend on the priors, such as $J^{T}C_{\epsilon }^{-1}J$. These terms are the same for all models and therefore cancel out in the subtraction. This yields the following result:

(17) $$\begin{array}{c} P_{i}-P_{F}=J_{i}^{T}C_{\epsilon }^{-1}J_{i}+\Pi_{i}-J_{f}^{T}C_{\epsilon }^{-1}J_{f}-\Pi_{F}=\Pi_{i}-\Pi_{F},\\ P_{i}=P_{F}+\Pi_{i}-\Pi_{F}. \end{array}$$

Following exactly the same procedure we obtain the conditional mean of the reduced model as a function of the mean of the full model and the priors for both models. To summarise, the post-hoc approach provides estimates of the parameters (means and precision) under the Laplace assumption for any reduced model that can be obtained by inverting only the full model:

(18) $$\begin{array}{c} P_{i}=P_{F}+\Pi_{i}-\Pi_{F}\\ \mu_{i}=C_{i}(P_{F}\mu_{F}+\Pi_{i}\eta_{i}-\Pi_{F}\eta_{F}). \end{array}$$

This method is exact for linear models (Friston and Penny, 2011). In the results section we test the validity of this approximation for bilinear deterministic DCMs. We compare the parameter estimates obtained with the post-hoc approach to the variational estimates obtained from optimising all models, using synthetic and real fMRI data. Finally, once the model evidence and parameters have been estimated for each model, *m*, using the optimised or post-hoc approximations, these estimates can then be used for model selection as described in the following section.

### 2.4 Bayesian model selection

The posterior model probability, *p*(*m*|*y*), can be obtained from the model evidence through Bayes’ rule:

(19) p(m|y)∝p(y|m)p(m),

where *p*(*m*) is the prior distribution over models. Selecting the optimal model corresponds to choosing the model *m* that maximises the posterior *p*(*m*|*y*). If no model is favoured a priori then *p*(*m*) is a uniform distribution, and the model with the highest posterior probability is also the model with the highest evidence, *p*(*y*|*m*) (Kass and Raftery, 1995).

Given two models, *m*_*i*_ and *m*_*j*_, we can compare these models using Bayes Factors (BFs), which are defined as the ratio of the corresponding model evidences. Equivalently, log-Bayes factors are given by differences in log-evidences:

(20) $$\ln B_{\mathrm{ij}}=\ln p(y|m_{i})-\ln p(y|m_{j})=F_{i}-F_{j}.$$

Bayes factors have been stratified into different ranges deemed to correspond to different strengths of evidence (Kass and Raftery, 1995). ‘Strong’ evidence, for example, corresponds to a BF of over 20 (log-BF over 3) in favour of model *m*_*i*_ when compared to model *m*_*j*_. Under uniform priors, Bayes’ rule gives:

(21) $$p(m_{i}|y)=\frac{1}{1+1/B_{\mathrm{ij}}},$$

and a posterior model probability greater than 0.95 is equivalent to a Bayes factor greater than 20.

In the following section we evaluate the methods described here with a synthetic and real fMRI dataset from an attention to visual motion paradigm.

## 3 Results

In this section we compare the optimised and post-hoc model evidences and parameter estimates with synthetic and real fMRI data.

The data were acquired by Buchel and Friston (1997) during an attention to visual motion paradigm. This dataset has been used to illustrate the post-hoc model selection approach on stochastic DCMs (Friston and Penny, 2011), as well as other methodologies from psychophysiological interactions (Friston et al., 1997) to Generalised Filtering (Friston et al., 2010). This dataset is publicly available on the SPM website (http://www.fil.ion.ucl.ac.uk/spm/). In this paper we use ‘DCM10’ as implemented in SPM8, revision 4010.

fMRI data were acquired from a normal subject with a 2 Tesla Magnetom VISION (Siemens, Erlangen) whole body MRI system, during a visual attention study. Contiguous multi-slice images were obtained with a gradient echo-planar sequence (TE = 40 ms; TR = 3.22 s; matrix size = 64 × 64 × 32, voxel size 3 × 3 ×3 mm). Four consecutive 100 scan sessions were acquired, comprising a sequence of ten scan blocks of five conditions. The first was a dummy condition to allow for magnetic saturation effects. In the second, *Fixation*, the subject viewed a fixation point at the centre of a screen. In an *Attention* condition, the subject viewed 250 dots moving radially from the centre at 4.7°/s and was asked to detect changes in radial velocity. In *No attention*, the subject was asked simply to view the moving dots. In a *Static* condition, the subject viewed stationary dots. The order of the conditions alternated between *Fixation* and visual stimulation (*Static*, *No Attention*, or *Attention*). In all conditions the subject fixated the centre of the screen. No overt response was required in any condition and there were no actual changes in the speed of the dots. The data were pre-processed and analysed using the conventional SPM analysis pipeline (http://www.fil.ion.ucl.ac.uk/spm/), as described in Buchel and Friston (1997).

For this work we chose three representative brain regions defined as clusters of contiguous voxels in an 8 mm sphere surviving an *F*-test for all effects of interest at *p* < 0.001 (uncorrected), using SPM. These regions are: the primary visual cortex (V1), [0, − 93, 18] mm in MNI space, the middle temporal visual area (V5), [− 36, − 87, − 3] mm, and the superior parietal cortex (SPC), [− 27, − 84, 36] mm (Buchel and Friston, 1997). The activity of each region was summarised with its principal eigenvariate to ensure an optimum weighting of contributions from each voxel within the region of interest (ROI).

### 3.1 Synthetic data

**Fig. 2:**
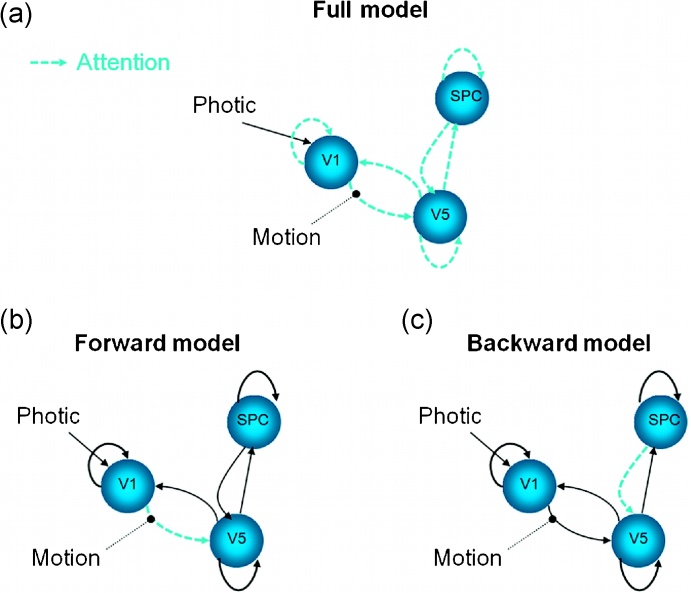
Model space: (a) Full model. In this model Attention modulates all the intrinsic connections and self-connections. This is the only model that needs to be inverted in order to estimate the evidence and parameters of all 2^7^ = 128 models, when using the post-hoc and Savage-Dickey approximations. The following models vary in which connections are modulated by Attention (dashed arrows). (b) True model from which synthetic data were generated. In this model Attention only modulates the connection from V1 to V5. Consequently, we call it the Forward model (as opposed to the Backward model). (c) Backward model: in this model Attention modulates the connection from SPC to V5.

*Model space.* Model space comprised 128 models. These models have full fixed connectivity (bidirectional connections) between V1 and V5 and between V5 and SPC (Fig. 2a). We allowed Motion to modulate only the connection from V1 to V5, but Attention was allowed to modulate any connection in the network, including the three self-connections (one for each region). In total we have 7 connections that can be modulated by Attention (3 self-connections + 4 intrinsic connections) resulting in 2^7^ = 128 different models. The full model (Fig. 2a) is the model for which Attention modulates all these 7 connections.

We note that we chose to specify different models by changing only modulatory parameters because these connections comprise the bilinear terms (*B* matrices) in Eq. (1). This way we can evaluate Eq. (18), which provides estimates for the reduced parameters based on the full model, under non-linear conditions.

We started by generating data from model 96 by integrating the DCM equations (Friston et al., 2003) and adding Gaussian noise corresponding to a Signal to Noise Ratio (SNR) of 2.6 (data and noise had a standard deviation of about.350 and 0.135, respectively, SNR = 0.350/0.135 = 2.59), as used in Friston et al. (2011). In this model, Attention only modulates the connection between V1 and V5. Therefore, we refer to this model as the Forward model (Fig. 2b). Fig. 2c shows another example model, in which Attention modulates the connection between SPC and V5. We refer to this model as the Backward model.

To obtain the model evidence and parameter estimates for all 128 models using the optimised approach we had to invert (optimise) all these models. The optimisation procedure took approximately 5 h in a 64-bit workstation. In comparison, for the post-hoc approach we only had to invert the full model, which took less than 2 min.

**Fig. 3:**
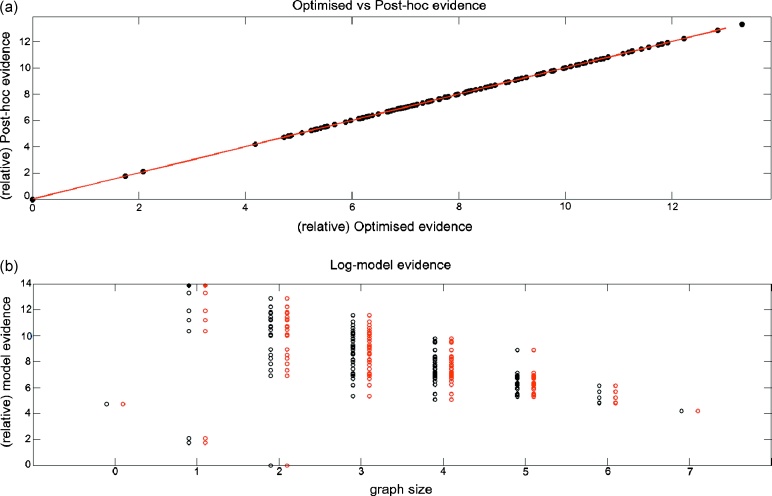
Synthetic data – model evidence: (a) Optimised log-model evidence (relative to worst model) versus post-hoc log-model evidence (128 synthetic models). (b) Same data but plotted as a function of graph size (number of edges or modulated connections). The red circles correspond to the post-hoc estimates, while the black correspond to the optimised approach. The full circles indicate the best models for each approximation. (For interpretation of the references to colour in this figure legend, the reader is referred to the web version of the article.)

*Model evidence.* Fig. 3a shows the optimised model evidence plotted against the post-hoc evidence for all 128 models. Here the evidence is relative to the worst model. As can be seen, the post-hoc measures correlate extremely well with the estimates obtained from optimising all models (they lie along the *y* = *x* line). The actual correlation value is almost 1 (*r* ≈ 1, *p*-value < 1*e*^−308^). Fig. 3b shows the relative evidences for the two approaches but as a function of graph size (number of edges). Again, the estimates for the model evidence obtained using the two approaches are extremely similar. Reassuringly, the true model (Forward model) has the highest log-evidence for both approximations and for the correct graph size (full circle): only one connection being modulated, in this case from area V1 to area V5.

**Fig. 4:**
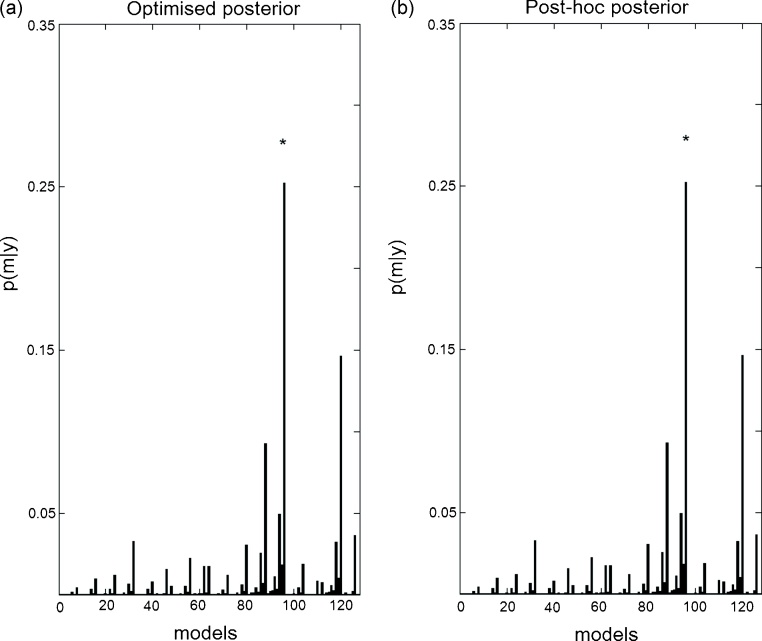
Synthetic data – Bayesian model selection: (a) Optimised model posteriors. The data were generated from model 96, Forward model (Fig. 2b) (marked by an asterisk, *). This model is also the best model for both approximations. (b) Post-hoc posterior probabilities. The backward model is model number 126 and the Bayes factor between the Forward and Backward model is 1.94 (as expected from Fig. 6).

Using the same synthetic data generated from model 96 (the Forward model in Fig. 2b) we looked at the model posterior probabilities for all 128 models. Again for the optimised approach we inverted all models, whilst for the post-hoc approach only the full model was inverted. As can be seen in Fig. 4, even though, as expected due to the number of models, the posterior mass is diluted over the models and no single model has very high probability, the true model (marked by the asterisk) has the highest posterior in both the optimised and post-hoc approaches.

**Fig. 5:**
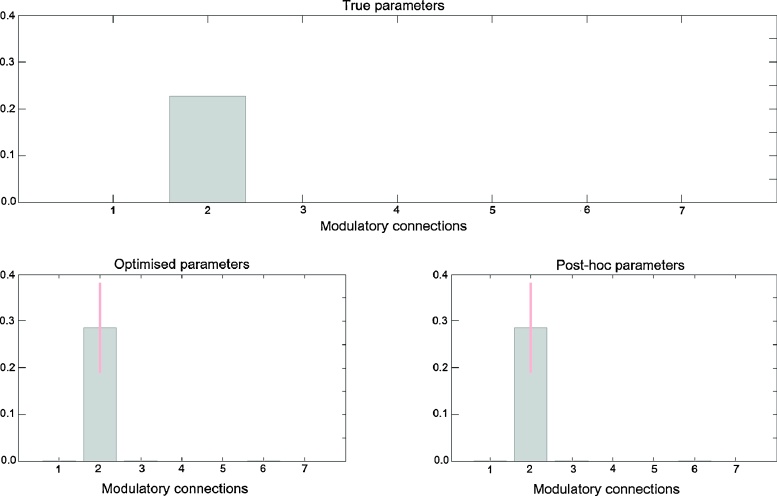
Synthetic data – parameter estimates: (a) True parameters from which the data were generated; Only the second parameter is modulated: forward connection from V1 to V5. (b) Optimised and post-hoc parameter estimates for the best model (Fig. 4). The error bars correspond to 95% confidence intervals. The parameters 1–7 (*x* axis) correspond to the 7 connections possibly modulated by Attention.

*Model parameters.* We then looked at the connectivity parameter estimates obtained with the optimised and post-hoc estimation approaches. Fig. 5a shows the true connection strengths that were used to generate the data, again from the same model (Forward model). We have 7 connections but only one of them (from V1 to V5) has a value different from zero. The second row of plots in Fig. 5 shows the parameter estimates (mean and 95% confidence intervals) obtained with the optimised and post-hoc approaches, respectively, corresponding to the best model identified previously (Fig. 4). As can be seen, both approaches identify the second parameter as being the only connection significantly different from zero. The true parameter value is 0.23 and both the optimised and post-hoc posterior means for this parameter are estimated as 0.29. The parameter estimates are summarised in Table 1.

These results show that, even though Eq. (18) is only an approximation in the case of non-linear models, it provides good estimates for bilinear DCMs.

*Signal-to-noise ratio.* The previous results have been obtained by generating data from one model and looking at how the different approaches to estimate the evidence and parameters compare using a fixed SNR similar to the SNR of the real fMRI dataset. This dataset comes from a block design paradigm and therefore has relatively high SNR. In this section we explore the behaviour of the two approaches for different values of SNR. To this end we performed two different model comparisons: (i) we generated data from the Forward model and compared this model to another model called the Backward model (Fig. 2); (ii) we generated data from the full model and compared this model to the Forward model described above. For both these comparisons we varied the SNR of the data from 0.35 to 3.35 in intervals of 0.1.

**Fig. 6:**
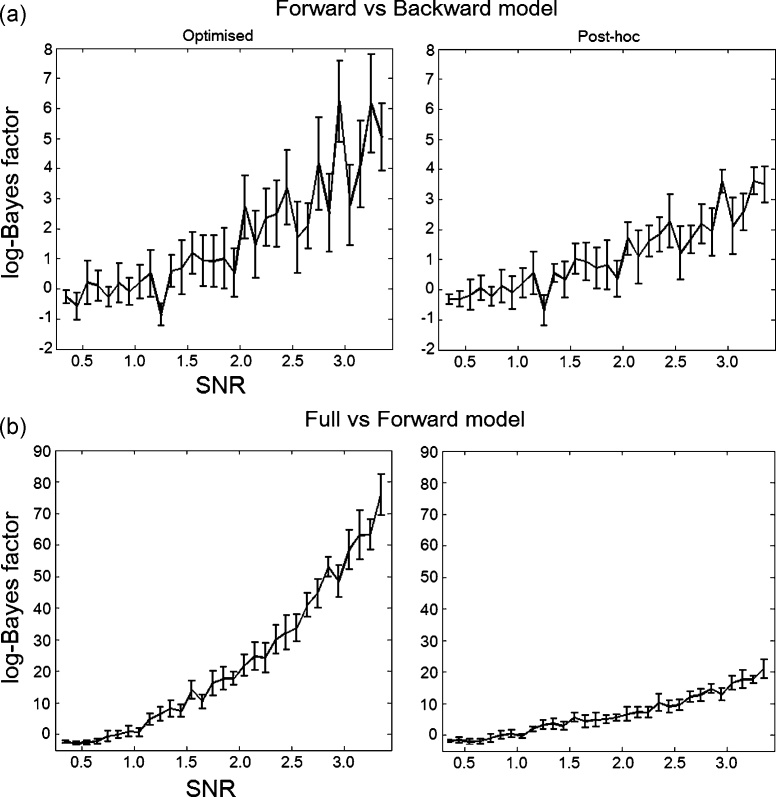
Signal-to-noise ratio – Bayes factors: (a) log-Bayes factors (between the forward and backward model) averaged over 10 repetitions of the same comparison (with 95% confidence intervals) as a function of the signal to noise ratio used to generate the data (from forward model); (b) log-Bayes factors (between the full and forward model) averaged over 10 repetitions of the same comparison (with 95% confidence intervals) as a function of the signal to noise ratio used to generate the data (from full model).

We also repeated the data generation, optimisation and model comparison 10 times for each SNR, in order to have 10 realisations of the same result. We then plotted (Fig. 6) the mean log-Bayes factor and 95% confidence intervals for each comparison as a function of the SNR. To obtain these results with the optimised approach we had to invert both the Forward and Backward models (first comparison), and Full and Forward models (second comparison) for each SNR and realisation. For the post-hoc approach we had only to invert the Full model for each SNR and repetition in both cases.

Fig. 6a shows that, as expected, the log-Bayes factors increase with higher SNR. However, our simulations suggest that the optimised approach seems to reach significant results (log-Bayes factor higher than 3) slightly faster than the post-hoc approach. The fact that the log-Bayes factors are positive (with increasing SNR) means that both methods are selecting the true model as the best model, with increasing confidence. One other thing to note is that the error bars are relatively smaller for the post-hoc approach, suggesting that the results for the optimised evidence are more inhomogeneous. At low SNR (below 1) the log-Bayes factors are close to zero with the error bars enclosing this number, as expected. In this case none of the methods select a winning model. However, for very low SNR (first two points) both methods seem to slightly prefer the backward model (BF < 1). This result might be due to the difficulty of estimating the models under very low SNR conditions, which can lead to inaccurate model selection results with both methods.

The results for the second comparison, where the true model is the full model (Fig. 6b), are very similar. The log-Bayes factors for the optimised approach increase significantly faster than the post-hoc approach, but the error bars are again slightly bigger. Here too the log-Bayes factors increase positively, which means that both methods are selecting the full model as the best model, even though this model is penalised for extra complexity. However, in the low SNR case (first 4 points, between 0.35 and 0.65) both methods seem to select the Forward model as the best model (negative Bayes factors). This means that in the almost complete absence of data (i.e. presence of high levels of noise), the full model is highly penalised and both model selection methods prefer the simpler hypothesis, the Forward model.

**Fig. 7:**
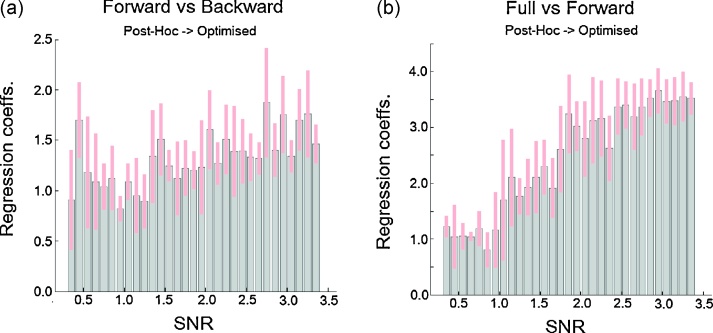
Signal-to-noise ratio – regression: (a) regression coefficients (and 95% confidence intervals) between optimised and post-hoc Bayes factors (comparing the forward model, true model, to the backward model) as a function of the signal to noise ratio; (b) regression coefficients (and 95% confidence intervals) between optimised and post-hoc Bayes factors (comparing the full model, true model, to the forward model) as a function of the signal to noise ratio.

We then regressed the post-hoc evidences onto the optimised evidences and looked at the regression coefficients. In Fig. 7 we plot these coefficients for both comparisons (Fig. 7a and b). As can be seen in the first case (Fig. 7a) the regression coefficients are all significantly different from zero and seem to slightly increase as a function of SNR. In the full versus forward model case (Fig. 7b) the results are very similar. Again all coefficients are significantly different from zero and increase as a function of SNR.

The previous results show that there is a linear relationship between the optimised and post-hoc measures (even in low SNR conditions) and that this relationship increases with increasing SNR.

In summary, the results obtained with synthetic data show that both approximations to the model evidence presented here yield similar results but the post-hoc approach reduced the computation time from a couple of minutes per model to a couple of seconds. In addition, even though the SNR of this dataset is relatively high (it is a block rather than event-related design) the post-hoc approach was also able to obtain the true model in lower SNR scenarios. The post-hoc estimates of the connectivity strengths were also very similar to the optimised and true estimates.

### 3.2 fMRI data

After testing the methods on synthetic data we turned to the fMRI dataset acquired by Buchel and Friston (1997). Here we used the time-series from the three brain regions V1, V5 and SPC for one subject as described above.

*Model space.* We used the same set of 128 models as defined before. The full model is the same full model used with synthetic data, in which Attention modulates all intrinsic connections between the three areas, as well as their three self-connections (Fig. 2a). In the optimised approach all 128 models were fitted to the fMRI signals. This took roughly the same amount of time to fit the synthetic data, since we used a similar signal to noise ratio to the real data. In the post-hoc approach only the full model was fitted to the fMRI data. Again this approach computed the evidences for all models in a few seconds.

**Fig. 8:**
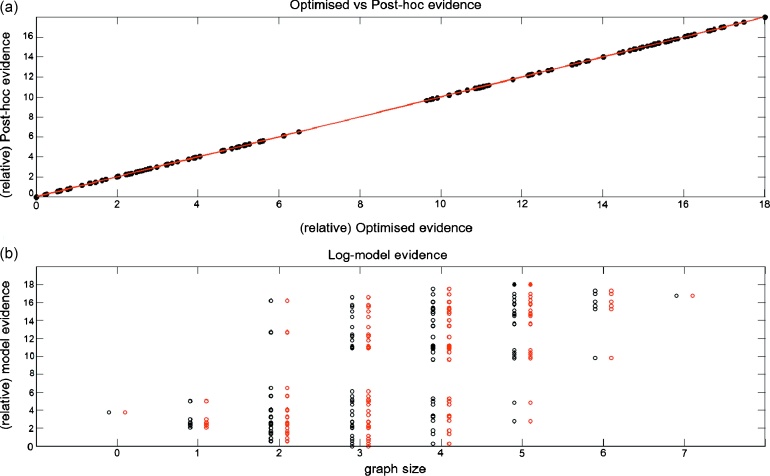
fMRI data – model evidence: (a) Optimised log-model evidence (relative to worst model) versus post-hoc log-model evidence (128 models). (b) Same data but plotted as a function of graph size (number of edges or modulated connections). The red circles correspond to the post-hoc estimates, while the black correspond to the optimised approach. The full circles indicate the best models for each approximation. (For interpretation of the references to colour in this figure legend, the reader is referred to the web version of the article.)

*Model evidence.* We plotted the post-hoc evidences against the model evidence obtained with the optimisation approach. As suggested by the results obtained with synthetic data, these measures correlate extremely well with the optimised evidences for this dataset (Fig. 8a), where *r* ≈ 1 (*p*-value < 1*e*^−308^). The best model identified by the optimised evidence is the same model (model 6) for the post-hoc approach. This model corresponds to a graph-size of 5, meaning that Attention modulates five connections (Fig. 8b): self-connections of V1 and V5, plus connections from V1 to V5, V5 to V1, and SPC to V5.

**Fig. 9:**
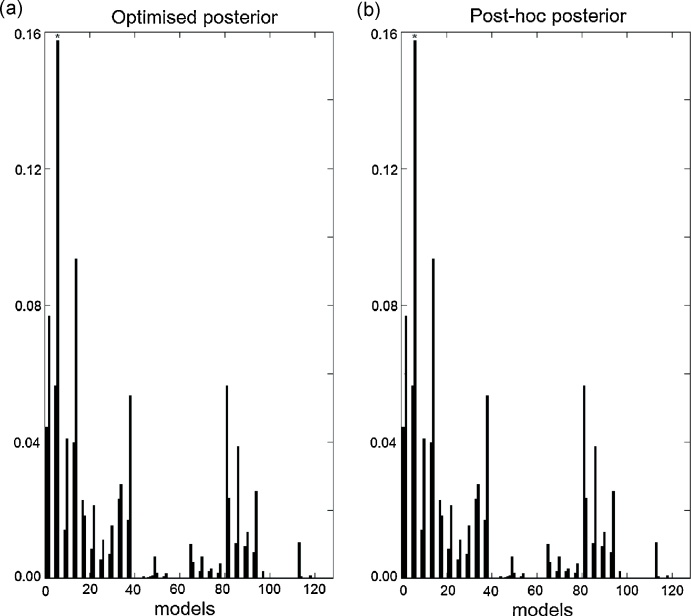
fMRI data – Bayesian model selection: (a) Optimised model posteriors. The best model, model 6, is marked by an asterisk, *. (b) Post-hoc model posteriors.

Fig. 9 shows the model posteriors obtained with both approaches for all 128 models using real fMRI data. As shown above (Fig. 8b), both methods identify model 6 as the best model with posterior probability close to 0.16.

**Fig. 10:**
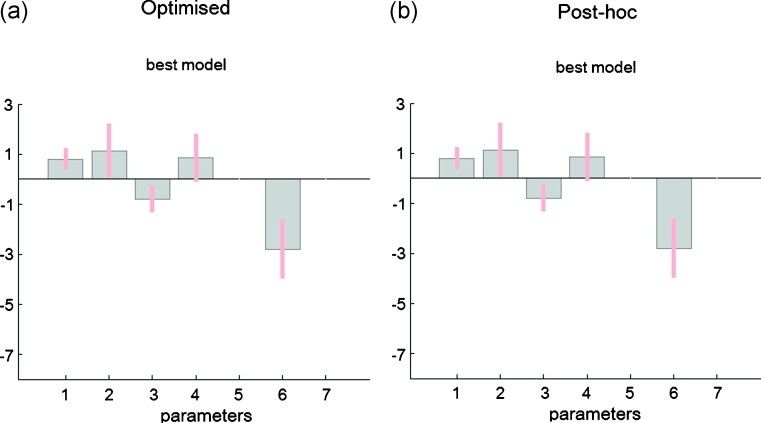
fMRI data – parameter estimates: Optimised and post-hoc parameter estimates for the best model, model 6 (Fig. 9). The error bars correspond to 95% confidence intervals. The parameters 1–7 (*x* axis) correspond to the 7 connections possibly modulated by Attention.

*Model parameters.* The parameter estimates (means and 95% confidence intervals) for the best model (model 6) are very similar for both approaches (Fig. 10). We can see that 5 of the total of 7 parameters seem to have values different than zero (although the error bars cross the zero line for the fourth parameter), as suggested by the best model by graph size in Fig. 8b (graph size 5). The values estimated for each connection are summarised in Table 1.

In summary, the results obtained with the real fMRI dataset are very similar to the ones obtained for synthetic data. Again the optimised and post-hoc methods provide very similar results both for the evidences and model parameters.

## 4 Discussion

In this paper we present and evaluate a recent approach, post-hoc approach (Friston and Penny, 2011), for estimating the model evidence and parameters of deterministic DCMs. This method offers substantial computational advantages to the variational free energy approach that is currently used (Friston et al., 2007).

Our results show very good agreement between optimised and post-hoc model evidences, for both synthetic and real fMRI data. This suggests that the post-hoc method offers valid estimates of the evidence with little computational cost. The post-hoc approach reduced the computation time needed to optimise and compare hundreds of models from several hours to a few minutes.

The reason why in some cases (e.g. when comparing the full versus a much smaller model (Fig. 6b)), the difference in log-evidences obtained by the post-hoc approach grows slower with SNR than the optimised approach, lies in the hyperparameter estimates. In the post-hoc approach the hyperparameters are assumed to be the same for all models (equal to the estimates for the full model), while in the optimised approach they are estimated for all models. When two similar models (e.g. forward versus backward model (Fig. 6a)) are compared, there are no significant differences in the behaviour of the two methods with SNR. This results from the fact that the hyperparameters estimated by the optimised approach do not vary significantly (*p*-value > 0.05 for all SNRs – full results not shown) between the models, and therefore both approaches obtain similar log-evidence differences even for high SNRs. However, when two very different models (e.g. full versus forward model) are compared, for high SNRs (e.g. 3.5), the difference in the hyperparameter estimates between models can be significant (*p*-value < 0.05 for SNRs higher than 2.5 – full results not shown). These estimates enter in the calculation of the optimised log-evidence and, for this reason, the optimised approach is more confident (bigger differences in log-evidences) for high SNRs than the post-hoc approach. This disparity, however, does not hinder the model comparisons since both approaches identify the same best (and true) model in the full range of SNRs studied.

The post-hoc method also provides estimates of the model parameters. Here we found that the post-hoc and optimised approaches yield very similar results. We have also shown that the post-hoc approach for estimating the model parameters, which is exact for linear models (assuming the hyperparameters are constant), seems to be a reasonably good approximation for non-linear models, such as DCMs. This results from the fact that by construction the models differ only in their priors. The model structure is the same for all models, and therefore their first-order approximation should also be the same. Although (bilinear) DCMs are not very non-linear, the degree of non-linearity should not affect the post-hoc estimates more than it affects the optimised approach. This is because the optimisation procedure that is implemented by the Variational Laplace algorithm, linearises the models at each iteration so as to obtain the posterior means and precisions.

As an aside, we note that we have also compared the post-hoc approximation to the model evidence, Eq. (12), to the Savage-Dickey approximation, Eq. (16), which is a special case of the former. As expected, these two measures yielded numerically identical results, including identical model posteriors. Moreover, when we regressed the Savage-Dickey Bayes-factors onto the post-hoc Bayes-factors for a wide range of SNRs (same as described in Section 3), we obtained regression coefficients equal to 1 for all SNRs.

Although the post-hoc approach is very computationally efficient, the number of possible models to compare can rapidly explode when considering networks with many regions and all possible connections between them. In this case, it might be impossible to compute the evidences and parameters for all models and one might have to sample the space of models. For instance, Pyka et al. (2011) use genetic algorithms to accelerate model selection of large numbers of DCMs. We are currently working on greedy searches and stochastic search algorithms that efficiently compute the post-hoc evidences and parameter estimates in arbitrarily large model spaces.

To conclude, our results provide evidence supporting the use of the post-hoc method proposed by Friston and Penny (2011) for model selection (and parameter inference) of bilinear deterministic DCMs.

## 5 Software

The software used for this work is available in the SPM8 software package (http://www.fil.ion.ucl.ac.uk/spm/).

## Figures and Tables

**Table 1:** Parameter estimates: posterior mean and 95% confidence intervals of the best model obtained with the optimised and post-hoc methods for synthetic and real data (first and second row of results for each connection, respectively). The subscript *op* means optimised, and *ph* means post-hoc.

Data	Connection	*μ*_*true*_	*μ*_*op*_	*μ*_*ph*_
*Parameter estimates*				
Synthetic	V1	0	0.00 ± 0.00	0.00 ± 0.00
Real		–	0.80 ± 0.45	0.80 ± 0.45
	V1 → V5	0.23	0.29 ± 0.10	0.29 ± 0.10
		–	1.14 ± 1.08	1.14 ± 1.08
	V5 → V1	0	0.00 ± 0.00	0.00 ± 0.00
		–	−0.79 ± 0.52	−0.79 ± 0.52
	V5	0	0.00 ± 0.00	0.00 ± 0.00
		–	0.85 ± 0.96	0.85 ± 0.96
	V5 → SPC	0	0.00 ± 0.00	0.00 ± 0.00
		–	0.00 ± 0.00	0.00 ± 0.00
	SPC → V5	0	0.00 ± 0.00	0.00 ± 0.00
		–	−2.79 ± 1.16	−2.79 ± 1.16
	SPC	0	0.00 ± 0.00	0.00 ± 0.00
		–	0.00 ± 0.00	0.00 ± 0.00

## Appendix A Priors

In this paper we used the priors from ‘DCM10’ as implemented in SPM8 (revision 4010). The priors on both the connectivity and haemodynamic parameters are assumed to be Gaussian and factorise over parameter types:

(A.1) p(θ|m)=p(A|m)p(B|m)p(C|m)p(h|m).

The priors on the fixed parameters (*A*) depend on the number of regions, *n*, to encourage stable dynamics. The priors on the fixed self-connections (*A*_*ii*_) are defined as follows:

(A.2) $$p(A_{\mathrm{ii}}|m)=N(-1/2,\sigma_{\mathrm{ii}}^{2}),$$

where $\sigma_{\mathrm{ii}}^{2}=1/(8\times n)$. In our case *n* = 3 regions, therefore *σ*_*ii*_ = 0.0417. The priors on the rest of the fixed parameters (*A*_*ij*_) are calculated as follows:

(A.3) $$p(A_{\mathrm{ij}}|m)=N\left(\frac{1}{\left(64*n\right)},\frac{8}{n}+\frac{1}{\left(8\times n\right)}\right).$$

In our case, this yields *p*(*A*_*ij*_|*m*) = *N*(0.0052, 2.7083). The rest of the connectivity parameters (modulatory and input parameters) have shrinkage priors:

(A.4) $$\begin{array}{c} p(B_{\mathrm{ij}}^{k}|m)=N(0,1),\\ p(C_{\mathrm{ij}}|m)=N(0,1). \end{array}$$

The unknown haemodynamic parameters are *h* = {*τ*_*s*_, *τ*_0_}. These are represented as

(A.5) $$\begin{array}{c} \tau_{s}=0.64\exp(\theta_{\tau_{s}})\\ \tau_{0}=2\exp(\theta_{\tau_{0}}), \end{array}$$

and have Gaussian priors:

(A.6) $$\begin{array}{c} p(\theta_{\tau_{s}})=N(\theta_{\tau_{s}};0,0.135)\\ p(\theta_{\tau_{0}})=N(\theta_{\tau_{0}};0,0.135). \end{array}$$

The overall prior density can then be written as *p*(*θ*|*m*) = *N*(*η*, *Σ*), where *η* and *Σ* are concatenations of the above means and variances.
